# Genetic insights into the relationship between immune cell traits and abnormal uterine bleeding: A Mendelian randomization study

**DOI:** 10.1097/MD.0000000000041609

**Published:** 2025-02-21

**Authors:** Wenting Ou, Pan Du, Xueling Bai, Yue He

**Affiliations:** a Department of Respiratory, Chongqing Emergency Medical Center, Chongqing University Central Hospital, Chongqing, China; b Department of Respiratory and Critical Care Medicine, West China Hospital, Sichuan University/West China School of Nursing, Sichuan University, Chengdu, Sichuan, China; c Nursing School of Zunyi Medical University, Zunyi, Guizhou, China; d Department of Nursing, Suining Central Hospital, Suining, Sichuan, China.

**Keywords:** abnormal uterine bleeding, amenorrhea, immune cell traits, Mendelian randomization, menorrhagia

## Abstract

Abnormal uterine bleeding (AUB) is an inflammatory response involving immune cells, but the relationship between immune cell traits and AUB is highly complex and still largely unclear. This study utilized genetic data from a genome-wide association study of European participants. Mendelian randomization (MR) analysis methods, including inverse variance weighted (IVW) as the primary approach, weighted median, MR Egger regression, and Mendelian randomization pleiotropy residual sum and outlier, were employed for forward and reverse analyses. Sensitivity analyses validated the stability and reliability of the results. The IVW method indicated a potential causal relationship between CD45 on granulocyte (odds ratio [OR] = 0.916, 95% CI: 0.880–0.954, *P* = 1.974 × 10^−5^) with a decreased risk of menorrhagia. Two immune cell traits with *P* values < .001 were worthy of attention, CD25 on naive-mature B cell (OR = 0.935, 95% CI: 0.901–0.970, *P* = 3.882 × 10^−4^) may be associated with a decreased risk of menorrhagia, while human leukocyte antigen DR on plasmacytoid dendritic cell (OR = 1.089, 95% CI: 1.038–1.143, *P* = 5.126 × 10^−4^) may be associated with an increased risk of amenorrhea. No reverse causation was observed. Sensitivity analysis suggested no heterogeneity or horizontal pleiotropy (*P* > .05). No immune cell traits associated with or potentially related to oligomenorrhea were found. This MR study highlights the complex relationship between immune cell traits and AUB, offering insights into AUB’s pathogenesis and potential biomarkers. Further clinical and in vitro validation is needed to assess these findings, with future research exploring immune modulation therapies for early diagnosis and personalized treatment.

## 1. Introduction

Abnormal uterine bleeding (AUB) is defined as irregularities in the volume, regularity, frequency, or duration of uterine bleeding.^[1]^ Globally, its prevalence ranges from 3% to 30%, with approximately one-third of women affected during their lifetime.^[2]^ AUB may impose a substantial economic burden, with annual direct costs ranging from $1 to $1.55 billion and indirect costs reaching $12 to $36 billion.^[3]^ In addition to its potential financial impact, AUB disrupts women’s work productivity, interpersonal relationships, and overall quality of life.^[4]^ A deeper understanding of the underlying mechanisms of AUB can facilitate the realization of personalized care.^[5]^

The occurrence of AUB is related to various factors, among which immune disorders may lead to AUB.^[6]^ One significant feature of menstruation is the inflammatory response involving immune cells.^[6]^

In the absence of pregnancy, a dynamic cell-to-cell dialogue between the endocrine and immune systems in the endometrium is crucial for ensuring effective shedding and repair of the endometrium.^[7]^ The human endometrium contains numerous immune cells, including T cells, macrophages/dendritic cells (DCs), natural killer cells, neutrophils, and mast cells.^[8]^ Uterine immune cells functionally adapt to changes throughout the menstrual cycle. For instance, macrophages play essential roles in inflammation regulation and promoting remodeling during menstruation.^[9]^ AUB is also an inflammatory process involving immune cells.^[10]^ Natural killer cells have been reported to disrupt endometrial blood vessel integrity and alter their function, contributing to irregular bleeding.^[11]^ Several observational studies have shown that AUB is more prevalent in patients with diseases closely associated with immune dysregulation, such as systemic lupus erythematosus, autoimmune thyroid diseases, and inflammatory bowel disease.^[12–14]^ This suggests that abnormalities in the immune system may play a significant role in the development of AUB. However, the relationship between immune cells and AUB is highly complex and not fully understood.

Causal inference often requires large-scale samples and robust evidence, but randomized controlled trials (RCTs) are frequently limited by ethical, financial, or technical barriers. Mendelian randomization (MR) uses genetic variants as instrumental variables (IVs) to simulate random allocation, effectively addressing confounding factors and reverse causation in traditional observational studies to some extent.^[15–17]^ Compared to traditional methods, MR offers clear advantages: exploring causal directions, minimizing confounding from environmental and behavioral factors, and leveraging large-scale genome-wide association study (GWAS) data for enhanced statistical power.^[15,17]^ This study employs MR to explore the link between immune cell traits and AUB, aiming to inform personalized care strategies.

## 2. Materials and methods

### 2.1. Study design

We used the 2-sample MR method to explore the potential relationship between immune cell traits and AUB. For 2-sample MR analysis, the single nucleotide polymorphisms (SNPs) used as IVs need to meet 3 assumptions: Relevance: IVs are closely associated with the exposure; Independence: IVs are independent of confounding factors; Exclusion restriction: IVs affect the outcome only through the exposure.

### 2.2. Data sources

The data for immune cell traits were obtained from the GWAS Catalog (https://www.ebi.ac.uk/gwas/), which provided detailed summary statistics (accession numbers GCST0001391 to GCST0002121), refer to Table S1, Supplemental Digital Content, http://links.lww.com/MD/O409. The data comprised a group of 3757 individuals from Sardinia, analyzing 539 immune traits, including 118 absolute cell counts, 192 relative counts, 389 surface antigen median fluorescence intensities, and 32 morphological parameters. This dataset also detailed various cell types, including myeloid cells, B cells, monocytes, TBNK (T cells, B cells, natural killer cells), DCs, and Treg panels.^[18]^

For AUB, we selected 3 phenotypes: menorrhagia, oligomenorrhea, and amenorrhea, based on the International Classification of Diseases, 10th Revision diagnoses from the latest release of data from FinnGen (https://r10.finngen.fi/).^[19]^ Specifically, menorrhagia was defined as “excessive and frequent menstruation with a regular cycle,” excluding postmenopausal bleeding, and included 23,458 cases and 111,583 controls. Oligomenorrhea was defined as “primary oligomenorrhea,” “secondary oligomenorrhea,” and “unspecified oligomenorrhea,” excluding ovarian dysfunction, and included 1150 cases and 111,583 controls. Amenorrhea was defined as “primary amenorrhea,” “secondary amenorrhea,” and “unspecified amenorrhea,” and included 2471 cases and 111,583 controls. For more detailed information on the data sources, refer to Table S1, Supplemental Digital Content, http://links.lww.com/MD/O409.

This MR study was conducted based on publicly available data and did not require ethical approval from an ethics committee.

### 2.3. Instrumental variables selection

The process of instrumental variable (IV) selection was conducted with meticulous attention to ensure validity and minimize bias. For forward MR analysis, where immune cell traits were considered as exposure, the significance threshold for IV was set at 1 × 10^−5^, considering the limited number of available SNPs. For reverse MR analysis, where AUB was considered as exposure, the significance level was adjusted to 5 × 10^−8^. We excluded SNPs that showed linkage disequilibrium, defined as SNPs with an *r*² >0.001 within a 10,000 kb window of the most significant SNPs in each cluster. To mitigate the risk of weak instrument bias, SNPs with an *F* statistic value <10 were excluded. Subsequently, SNPs were screened using PhenoScanner3 to exclude those associated with horizontal pleiotropy and potential confounders, such as polycystic ovary syndrome, contraceptive use, coagulation disorders, and obesity.

### 2.4. MR analysis

We employed various MR analysis methods, including inverse variance weighted (IVW), weighted median, MR Egger regression, and Mendelian randomization pleiotropy residual sum and outlier (MR-PRESSO). Due to the high statistical efficiency of the IVW method, its ability to handle multiple SNPs, integration of information from all SNPs, strong robustness, and wide applicability, the IVW method was selected as the primary approach for reliably estimating causal effects.^[20–22]^

Sensitivity analyses were further conducted: Heterogeneity test: Cochrane’s *Q* value was used to assess the heterogeneity of SNP estimates in MR analysis. If heterogeneity was present (*P* < .05), an IVW random effects model was used for correction; otherwise, a fixed effects model was used; Pleiotropy test: MR-Egger regression was used to identify potential pleiotropy. A nonsignificant MR-Egger intercept (*P* > .05) indicated a lack of horizontal pleiotropy.^[23]^ If pleiotropy was present, the MR-PRESSO method was used to detect outliers, and MR analysis was repeated after removing outliers until no pleiotropy remained; Leave-one-out analysis was performed to investigate the potential impact of individual SNPs on the relationship between immune cell traits and AUB.^[24]^

All analyses were performed using R software, version 4.3.1. Using the Bonferroni correction to adjust for multiple testing, we set the significance threshold to a *P* value lower than .05/(731 × 3), that is, *P* < 2.280 × 10^−5^.

## 3. Results

### 3.1. The relationship between immune cell traits and menorrhagia

**Figure 1.**
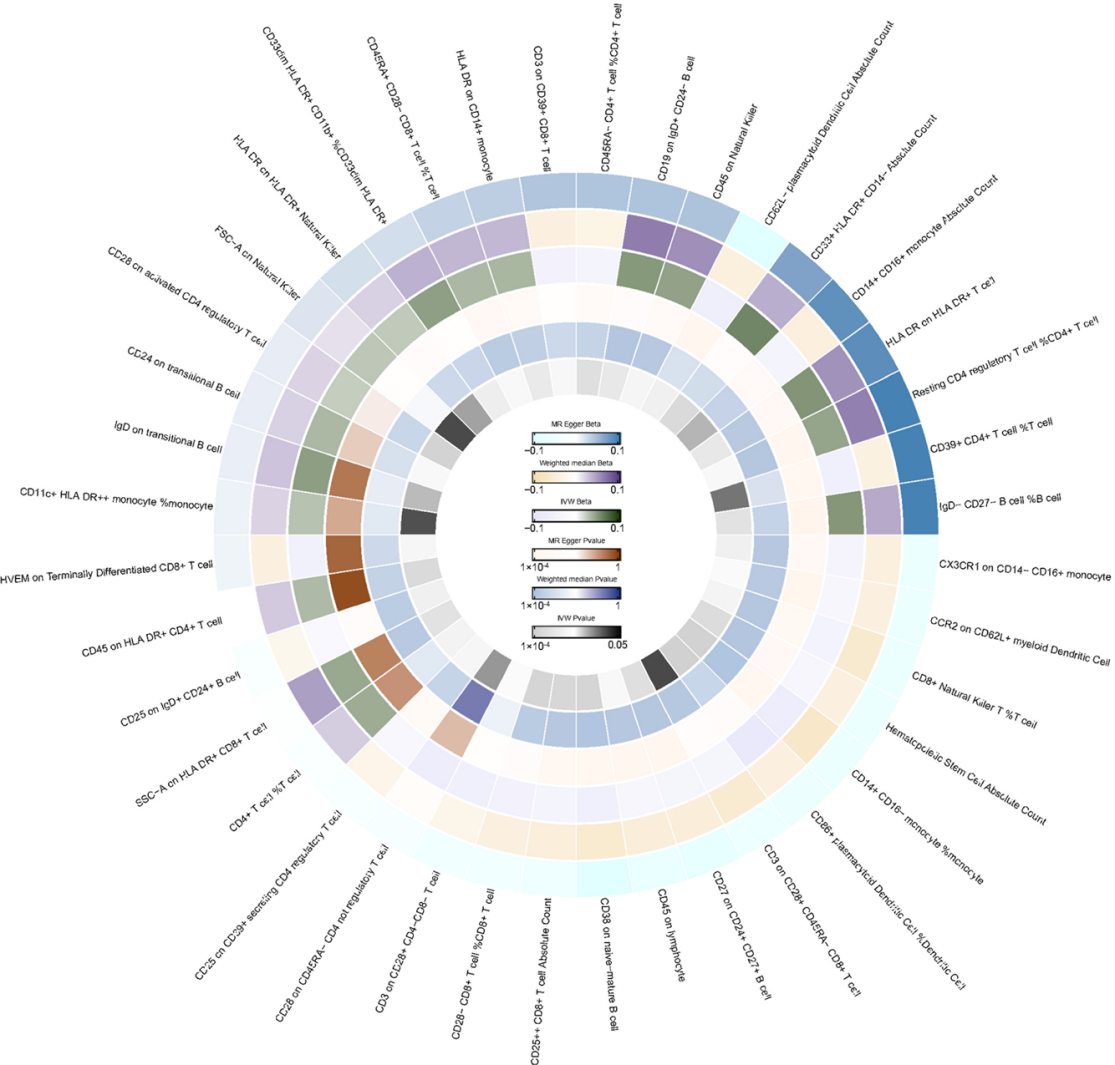
IVW analysis: relationship between 40 immune cell traits and menorrhagia (*P* < .05). IVW = inverse variance weighted.

**Figure 2.**
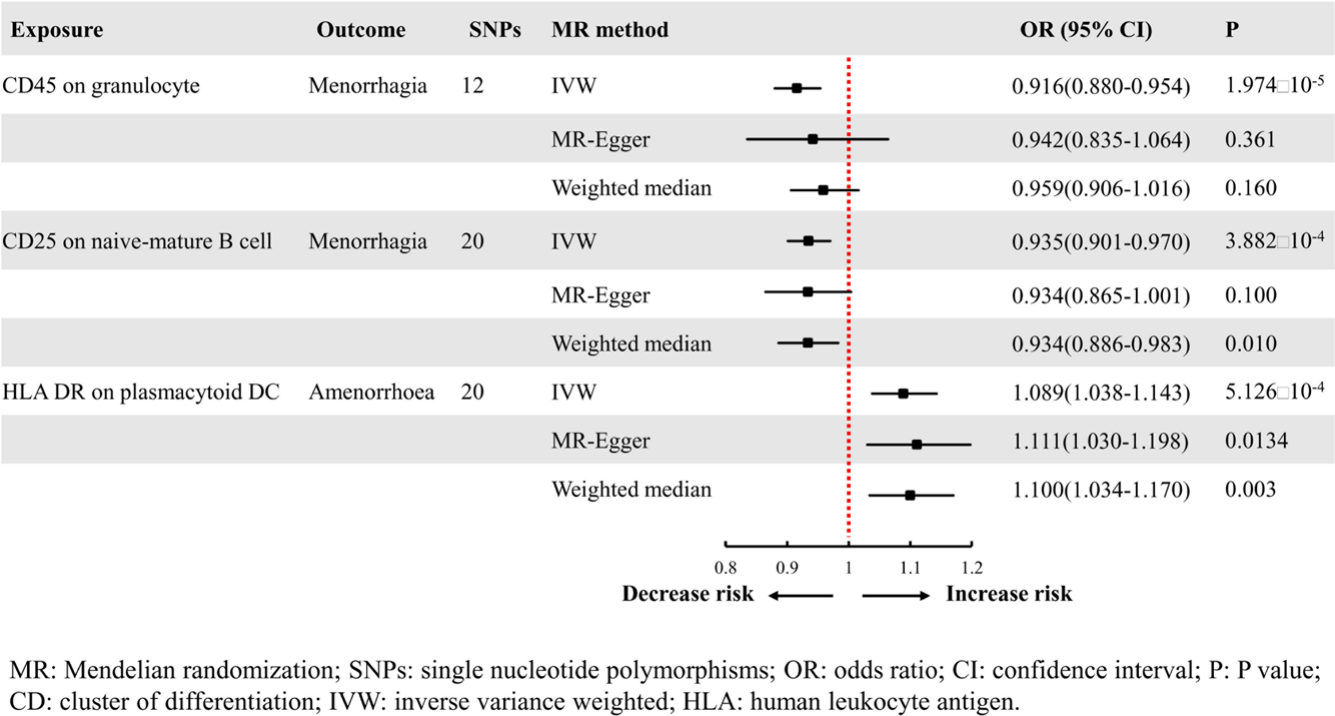
MR analysis results MR analysis results (immune cell traits with menorrhagia and amenorrhea). CI = confidence interval, CD = cluster of differentiation, HLA = human leukocyte antigen, IVW = inverse variance weighted, OR = odds ratio, SNP = single nucleotide polymorphisms.

In the relationship between immune cell traits and menorrhagia, we found 40 immune cell traits with a *P* value of <.05 using the IVW method, as shown in Figure 1. Notably, CD45 on granulocyte (IVW: odds ratio [OR] = 0.916, 95% CI: 0.880–0.954, *P* = 1.974 × 10^−5^; MR Egger: OR = 0.942, 95% CI: 0.835–1.064, *P* = .361; weighted median: OR = 0.959, 95% CI: 0.906–1.016, *P* = .160) was associated with a potential decreased risk of menorrhagia. Additionally, CD25 on naive-mature B cells, which showed a very low *P* value in the IVW analysis (IVW: OR = 0.935, 95% CI: 0.901–0.970, *P* = 3.882 × 10^−4^; MR Egger: OR = 0.934, 95% CI: 0.865–1.001, *P* = .100; weighted median: OR = 0.934, 95% CI: 0.886–0.983, *P* = .010), was possibly associated with a decreased risk of menorrhagia. Refer to Figure 2.

In further sensitivity analysis, Cochrane’s *Q* test indicated no evidence of heterogeneity for CD45 on granulocyte (*P* = .178) and CD25 on naive-mature B cell (*P* = .709). The MR-Egger regression test showed no evidence of horizontal pleiotropy for CD45 on granulocyte (intercept = −0.006, *P* = .630) and CD25 on naive-mature B cell (intercept = −1.863 × 10^−4^, *P* = .980). MR-PRESSO results indicated insufficient evidence of outliers and horizontal pleiotropy for CD45 on granulocyte (Global_test_pval = 0.254) and CD25 on naive-mature B cell (Global_test_pval = 0.739), as shown in the Table S1, Supplemental Digital Content, http://links.lww.com/MD/O409. The leave-one-out method suggested the robustness of the MR results (Figs. S1 and S2, Supplemental Digital Content, http://links.lww.com/MD/O410).

Using the IVW method, reverse MR analysis was conducted for the above 2 immune cell traits and menorrhagia. The results indicated that there was no reverse relationship between CD45 on granulocyte (*P* = .694) and CD25 on naive-mature B cell (*P* = .056) with menorrhagia.

### 3.2. The relationship between immune cell traits and oligomenorrhea

**Figure 3.**
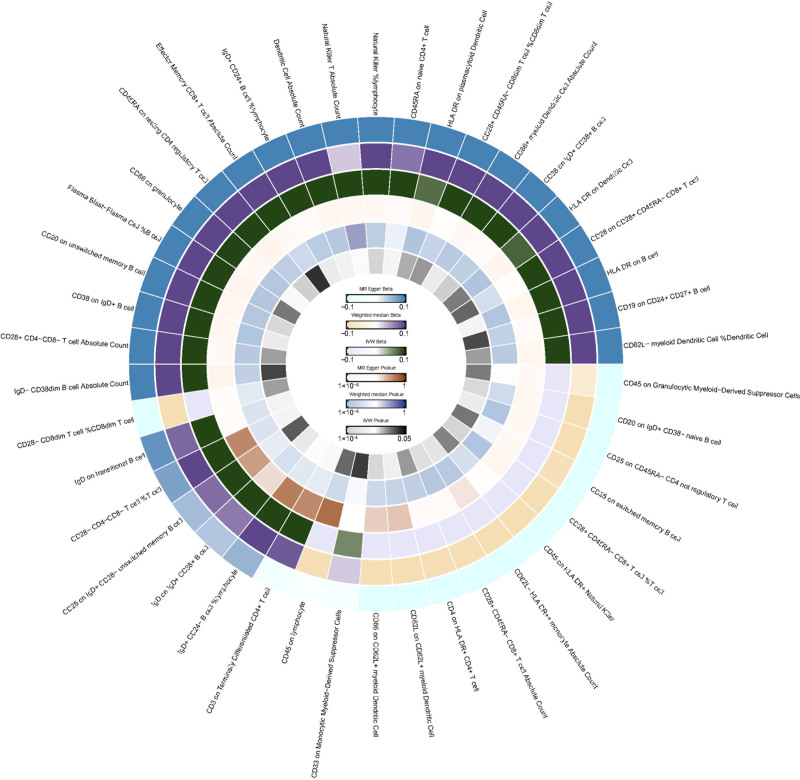
IVW analysis: relationship between 42 immune cell traits and oligomenorrhea (*P* < .05). IVW = inverse variance weighted.

In the relationship between immune cell traits and oligomenorrhea, we found that 42 immune cell traits had a *P* value of <.05 using the IVW method, as shown in Figure 3. Among them, the trait with the smallest *P* value was CD28^+^ CD45RA^−^ CD8^+^ T cell %T cell (IVW: OR = 0.824, 95% CI: 0.727–0.933, *P* = 2.325 × 10^−3^). Therefore, we did not find any immune cell traits related to oligomenorrhea.

### 3.3. The relationship between immune cell traits and amenorrhea

**Figure 4.**
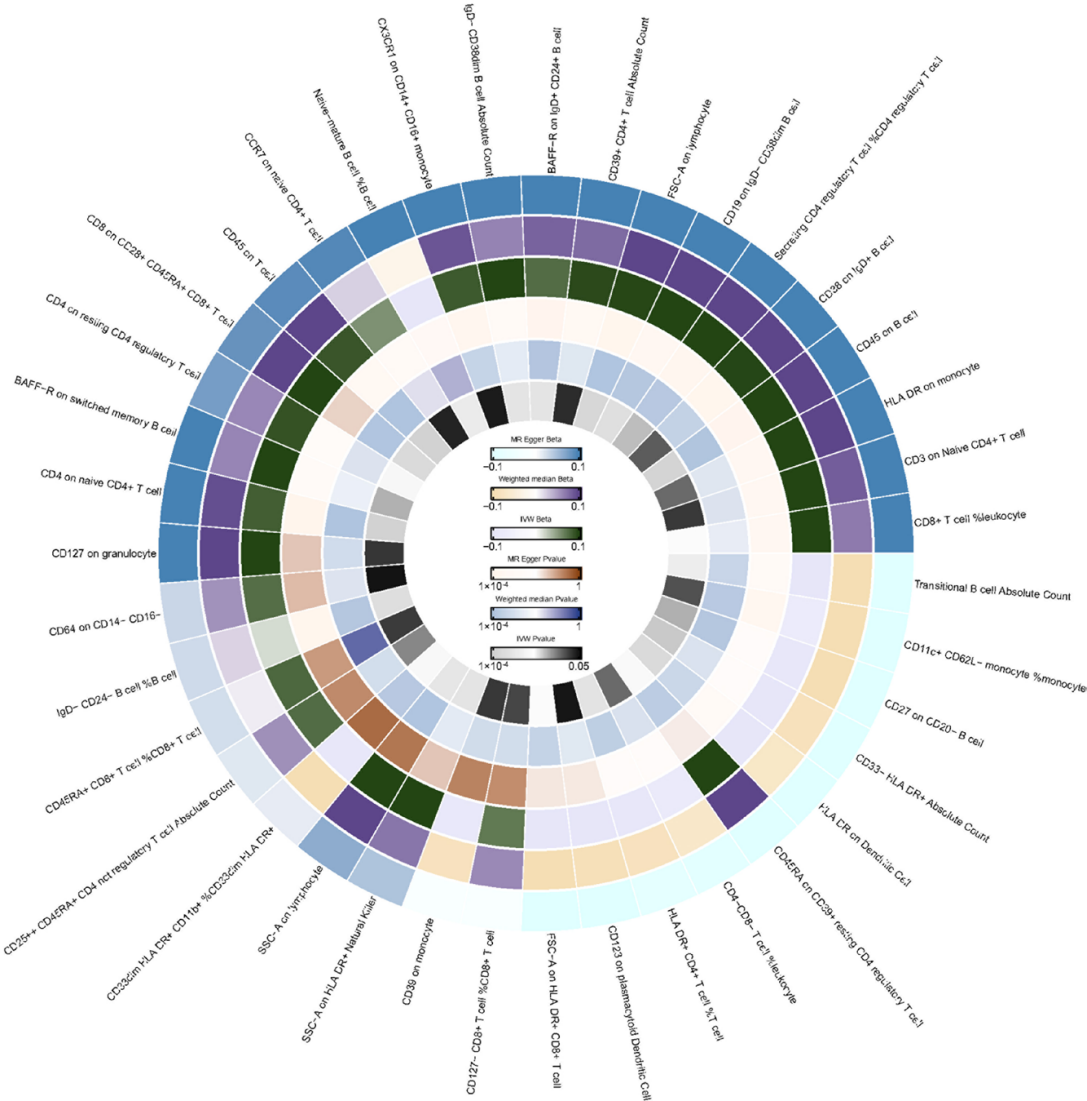
IVW analysis: relationship between 39 immune cell traits and amenorrhea (*P* < .05). IVW = inverse variance weighted.

In the relationship between immune cell traits and amenorrhea, we found that 39 immune cell traits had a *P* value of <.05 using the IVW method, as shown in Figure 4. We did not find any immune cell traits causally associated with amenorrhea. However, it is worth noting that among these traits, human leukocyte antigen (HLA) DR on plasmacytoid DC had the lowest and most significant *P* value (IVW: OR = 1.089, 95% CI: 1.038–1.143, *P* = 5.126 × 10^−4^; MR Egger: OR = 1.111, 95% CI: 1.030–1.198, *P* = .0134; weighted median: OR = 1.100, 95% CI: 1.034–1.170, *P* = .003), suggesting a possibly association with increased risk of amenorrhea, refer to Figure 2. Sensitivity analysis showed no heterogeneity (*P* = .848) according to Cochrane’s *Q* test, no horizontal pleiotropy (intercept = −0.010, *P* = .507) according to MR-Egger regression test, and no outliers or horizontal pleiotropy (*P* = .884) according to MR-PRESSO results, as shown in the Table S1, Supplemental Digital Content, http://links.lww.com/MD/O409. The leave-one-out method indicated the robustness of the MR results (Fig. S3, Supplemental Digital Content, http://links.lww.com/MD/O410).

Since only 2 SNPs were identified as IVs when amenorrhea was treated as the exposure, we did not conduct a reverse MR analysis.

## 4. Discussion

Using publicly available genetic data and the MR method, we investigated the potential causal relationship between immune cell traits and AUB. The results showed that CD45 on granulocyte was potentially causally associated with a reduced risk of menorrhagia (*P* = 1.974 × 10^−5^). Additionally, 2 immune cell traits with *P* < .001 (IVW) are noteworthy, suggesting possible associations: CD25 on naive-mature B cell may be associated with a reduced risk of menorrhagia, and HLA DR on plasmacytoid DC may be associated with an increased risk of amenorrhea.

CD45 is a protein tyrosine phosphatase expressed on leukocytes and plays a critical role in immune cell signaling and function.^[25]^ Starkey et al^[26]^ observed that CD45^+^ leukocytes reach their peak concentration at the onset of menstruation, highlighting their importance in menstrual physiology. These cells, particularly granulocytes like neutrophils, basophils, and eosinophils, exhibit multifunctional roles. Neutrophils, the predominant granulocytes, may enhance tissue repair through several mechanisms. It has been reported that CD45 can modulate or enhance the stimulation and function of human neutrophils mediated by FcγR.^[27]^ Lee et al^[28]^ pointed out that neutrophil-derived elastase may upregulate the expression and release of transforming growth factor-β1 gene, which can promote the repair of damaged endometrium,^[29]^ thereby reducing endometrial bleeding. Neutrophils may also secrete vascular endothelial growth factor (VEGF) and increase the bioavailability and bioactivity of VEGF by secreting matrix metalloproteinase 9.^[30,31]^ VEGF is crucial in endometrial angiogenesis and especially important for endometrial repair, particularly during the processes of angiogenesis and re-epithelialization.^[32,33]^ VEGF promotes the migration and differentiation of vascular smooth muscle cells by inducing the expression of platelet-derived growth factor and epidermal growth factor in endothelial cells.^[34]^ Graubert et al^[35]^ believe that the regulation of VEGF receptors by epidermal growth factor in the late menstrual phase helps initiate endometrial angiogenesis and endothelial repair. VEGF may also induce coagulation by stimulating the expression of tissue factor,^[36]^ reducing uterine bleeding. A study has shown that women with menorrhagia have significantly lower concentrations and reduced activity of VEGF, which may impair endometrial repair and early angiogenesis.^[37]^ Basophils and eosinophils also contribute by producing VEGF, supporting angiogenesis and endometrial integrity.^[38]^ In addition, CD45^+^ granulocytes may help reduce excessive endometrial bleeding by clearing endometrial cellular debris and preventing excessive inflammation. These potential mechanisms highlight the possible role of CD45^+^ granulocytes in reducing the risk of menorrhagia and emphasize their overall involvement in maintaining menstrual health.

Naive-mature B cells have matured but have not yet been activated, they are ready to be activated at any time. Upon activation, the expression of CD25 (the α chain of the interleukin [IL]-2 receptor) on B cells increases, producing a high-affinity IL-2 receptor and responding to low concentrations of IL-2.^[39]^ Human IL-2 through surface receptors similar to those on activated T cells, promotes the proliferation of activated B cells.^[40]^ Naive-mature lymphocytes are capable of undergoing homeostatic proliferation in response to lymphocyte-deficient environments, thereby maintaining lymphocyte numbers.^[41]^ This function may play a vital role during the menstrual cycle by helping maintain immune balance and reducing the risk of endometrial infections or other disruptions. Studies have also shown that B cells possess the ability to secrete VEGF to promote angiogenesis.^[42,43]^ Therefore, CD25 on naive-mature B cells may help lower the risk of menorrhagia by maintaining immune homeostasis and supporting vascular repair. However, further studies are needed to confirm this association and elucidate the underlying mechanisms.

Human leukocyte antigen (HLA): DR is a major histocompatibility complex II molecule that primarily presents antigens to CD4^+^ T cells. The HLA-DR molecules on the surface of DC are crucial for initiating specific immune responses. Plasmacytoid DC (pDC) expresses low levels of major histocompatibility complex II, which are upregulated upon activation.^[44]^ Activated pDCs induce the proliferation of T cell populations^[45]^ (Reference 2). By upregulating HLA-DR, pDCs more effectively present antigens to CD4^+^ T cells. CD4^+^ T cells are able to produce IL-10.^[46]^ Mammalian studies indicate that IL-10 inhibits VEGF induced angiogenesis.^[47]^ T helper 1 (Th1) cells secrete interferon-γ, suppressing the expression of VEGF.^[48]^ It has been reported that VEGF is an important regulator of follicle development and selection in mammals, determining follicular growth.^[49]^ Thus, the expression of HLA-DR on pDCs may play a role in modulating the immune response and angiogenesis, potentially contributing to the development of amenorrhea. However, further research is needed to fully understand the mechanisms behind this process and its clinical implications.

To the best of our knowledge, this is the first study applying MR methods to explore the relationship between immune cell traits and AUB. Our conclusions are drawn with careful consideration of horizontal pleiotropy. However, limitations exist. First, the data is sourced from European ancestry populations, lacks age stratification, and does not account for medication use (e.g., hormones or anticoagulants), potentially affecting the generalizability. Second, Determining the specific timing of circulating immune cell traits is challenging, making it difficult to confirm whether blood sampling occurred before, during, or after menstruation, potentially leading to bias in MR results. Third, the absence of an association between immune traits and oligomenorrhea may be due to the small sample size. Moreover, although our study provides valuable insights, it lacks in vitro experimental validation. Future laboratory research will be essential to further confirm these findings and explore the underlying biological mechanisms. Future research should also include larger sample sizes, more comprehensive Mendelian randomization designs, and clinical trials to thoroughly examine the relationship between immune cell traits and abnormal uterine bleeding.

## 5. Conclusion

In conclusion, our in-depth exploration of the relationship between immune cell characteristics and abnormal uterine bleeding (AUB) reveals the complex interaction between the 2. Our study offers new insights into the pathogenesis of AUB and suggests potential biomarkers and therapeutic targets. However, further validation through larger clinical studies and in vitro experiments is needed to assess the clinical relevance of these findings. Future research combining larger sample sizes, clinical data, and laboratory studies can further explore the role of immune cells in AUB, providing new directions for early diagnosis and personalized treatment. This may offer valuable insights for immune modulation therapies in AUB.

## Acknowledgments

We express our unreserved appreciation to all the international research groups or consortia from whom the GWAS data analyzed in this study were sourced.

## Author contributions

**Data curation:** Wenting Ou.

**Conceptualization:** Yue He.

**Formal analysis:** Wenting Ou.

**Investigation:** Pan Du.

**Methodology:** Wenting Ou.

**Project administration:** Xueling Bai.

**Resources:** Pan Du.

**Software:** Xueling Bai.

**Supervision:** Yue He.

**Validation:** Pan Du.

**Visualization:** Xueling Bai.

**Writing – original draft:** Wenting Ou, Pan Du.

**Writing – review & editing:** Xueling Bai, Yue He.

## Supplementary Material


