# Supplementary material for: Genetic insights into the relationship between immune cell traits and abnormal uterine bleeding: A Mendelian randomization study
Source: Medicine (Baltimore). 2025 Feb 21;104(8):e41609. doi: 10.1097/MD.0000000000041609 (PMC11856939; doi:10.1097/MD.0000000000041609)

Supplementary Figure 1. MR leave-one-out sensitivity analysis for ‘CD45 on granulocyte’ on ‘Menorrhagia’

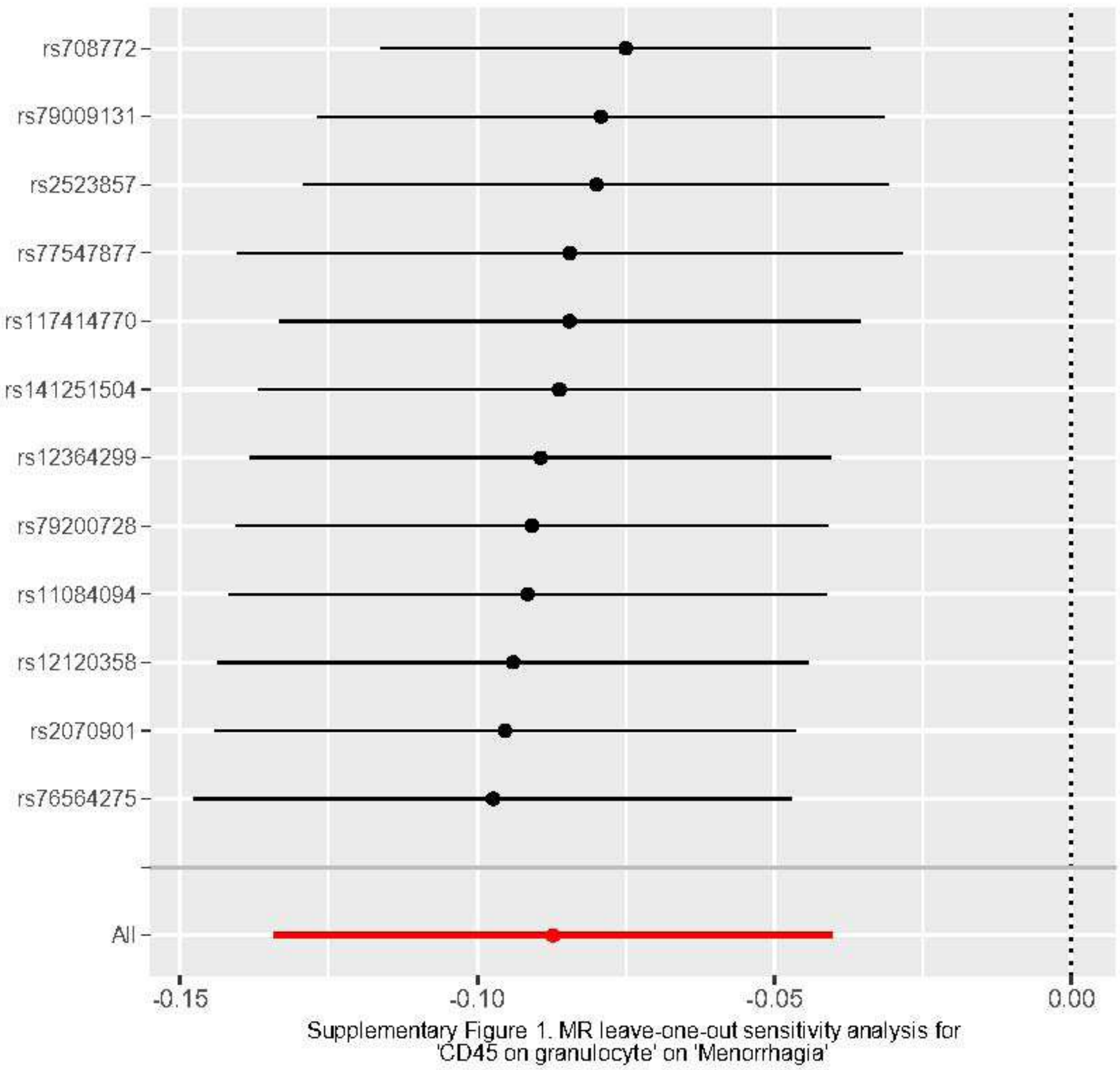

Supplementary Figure 2. MR leave-one-out sensitivity analysis for ‘CD25 on naive-mature B cell' on ‘Menorrhagia’

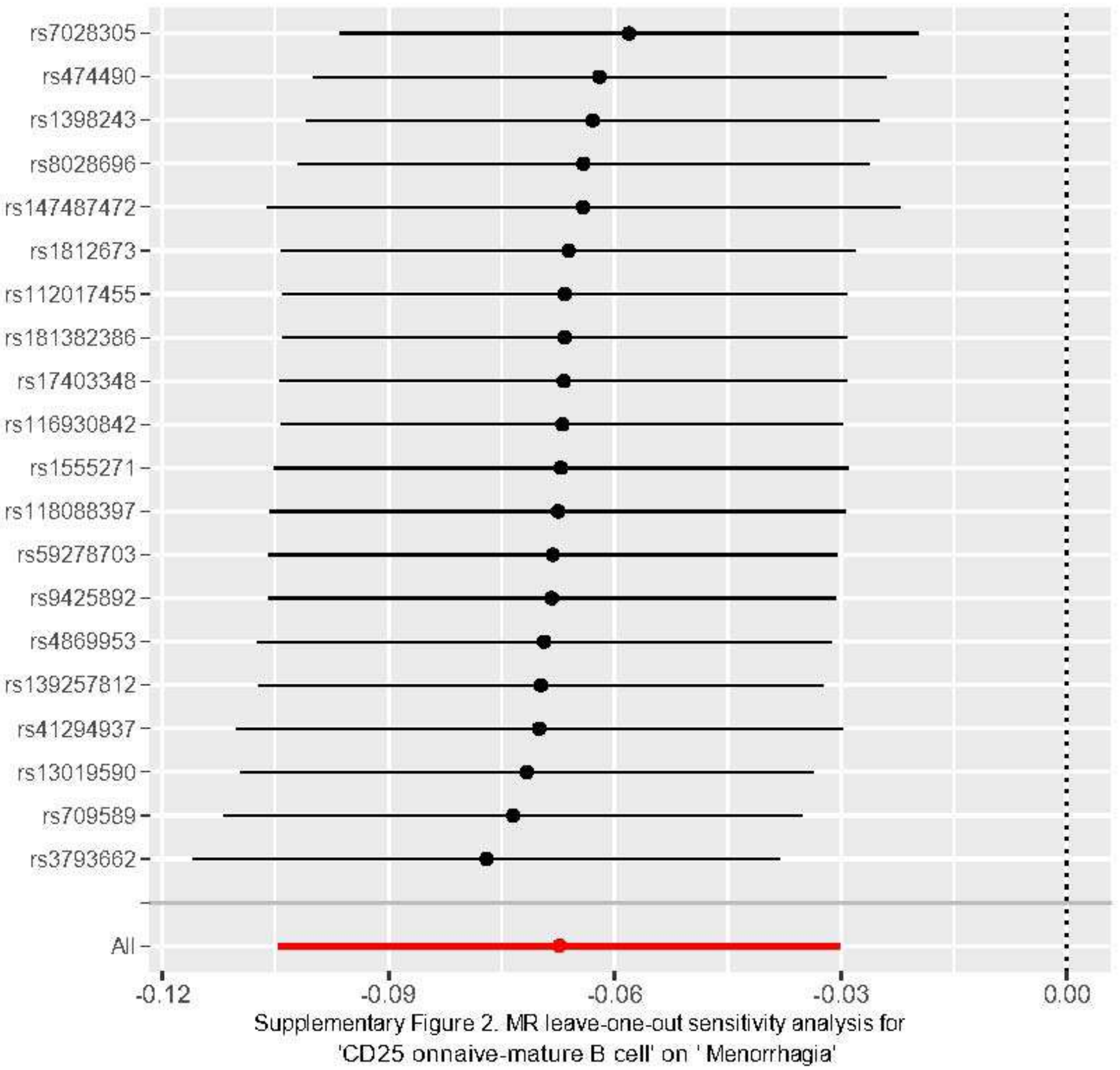

Supplementary Figure 3. MR leave-one-out sensitivity analysis for ‘HLA DR on plasmacytoid Dendritic Cell’ on ‘Amenorrhoea’

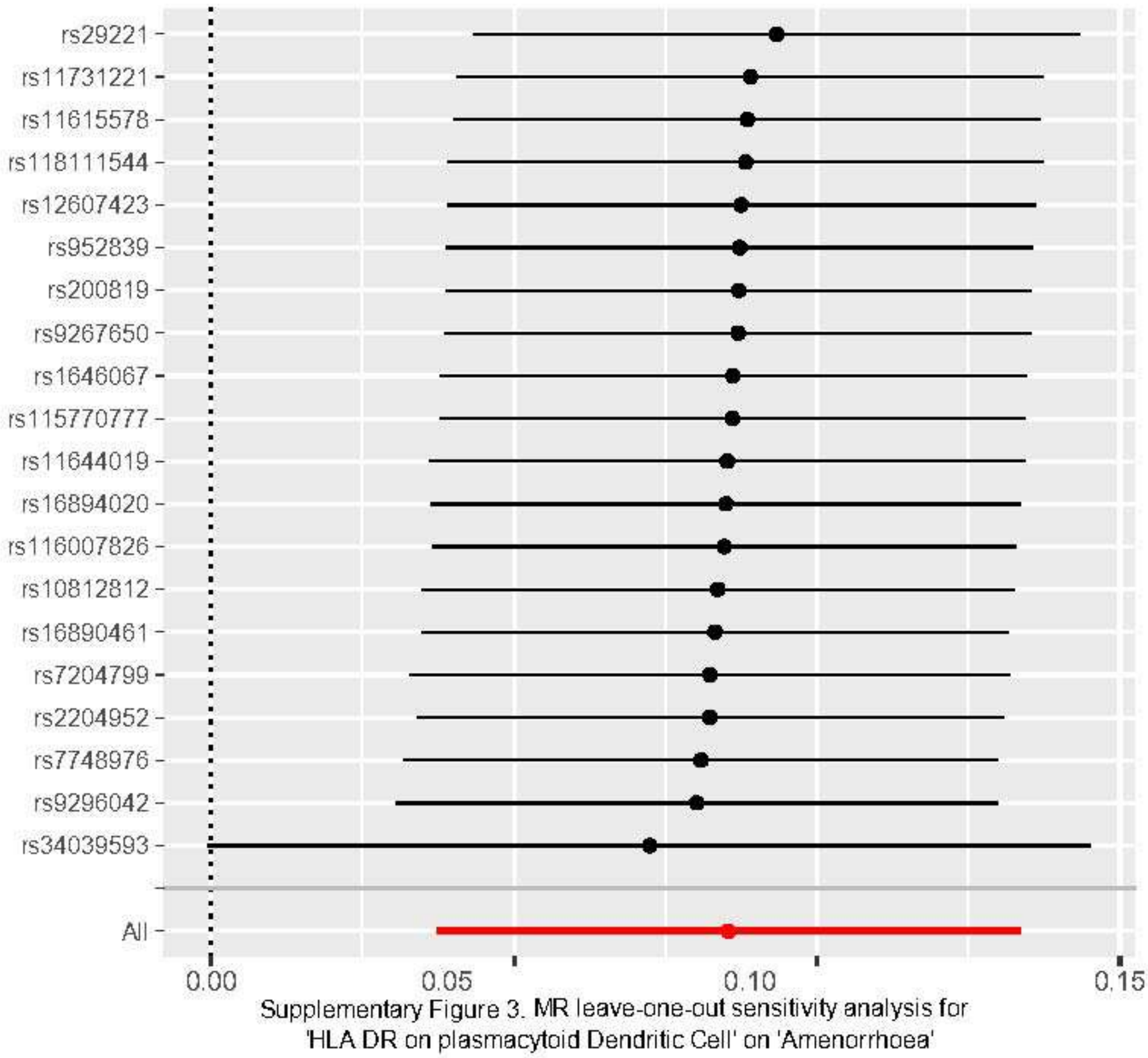

Supplement: Supplementary file 2 [file medi-104-e41609-s002.pdf]
